# Visualizing the Synaptic and Cellular Ultrastructure in Neurons Differentiated from Human Induced Neural Stem Cells—An Optimized Protocol

**DOI:** 10.3390/ijms21051708

**Published:** 2020-03-02

**Authors:** Philipp Capetian, Lorenz Müller, Jens Volkmann, Manfred Heckmann, Süleyman Ergün, Nicole Wagner

**Affiliations:** 1 Department of Neurology, University Hospital Würzburg, 97080 Würzburg, Germany; 2 Institute of Physiology, Department of Neurophysiology, University of Würzburg, 97070 Würzburg, Germany; 3 Institute of Anatomy and Cell Biology, University of Würzburg, 97070 Würzburg, Germany

**Keywords:** transmission electron microscopy, human neurons, induced neural stem cells, synapse, synaptic vesicles, high contrast

## Abstract

The size of the synaptic subcomponents falls below the limits of visible light microscopy. Despite new developments in advanced microscopy techniques, the resolution of transmission electron microscopy (TEM) remains unsurpassed. The requirements of tissue preservation are very high, and human post mortem material often does not offer adequate quality. However, new reprogramming techniques that generate human neurons in vitro provide samples that can easily fulfill these requirements. The objective of this study was to identify the culture technique with the best ultrastructural preservation in combination with the best embedding and contrasting technique for visualizing neuronal elements. Two induced neural stem cell lines derived from healthy control subjects underwent differentiation either adherent on glass coverslips, embedded in a droplet of highly concentrated Matrigel, or as a compact neurosphere. Afterward, they were fixed using a combination of glutaraldehyde (GA) and paraformaldehyde (PFA) followed by three approaches (standard stain, Ruthenium red stain, high contrast en-bloc stain) using different combinations of membrane enhancing and contrasting steps before ultrathin sectioning and imaging by TEM. The compact free-floating neurospheres exhibited the best ultrastructural preservation. High-contrast en-bloc stain offered particularly sharp staining of membrane structures and the highest quality visualization of neuronal structures. In conclusion, compact neurospheres growing under free-floating conditions in combination with a high contrast en-bloc staining protocol, offer the optimal preservation and contrast with a particular focus on visualizing membrane structures as required for analyzing synaptic structures.

## 1. Introduction

Neuronal synapses relay and transfer signals between cells and are key components of neural processing. The size of their sub-components (transmitter-filled vesicles, active zone, synaptic cleft, pre- and postsynaptic membranes) fall below the diffraction limits of visible light and thus conventional microscopy. Despite the advent of “superresolution” light microscopic techniques (e.g., direct stochastic optical reconstruction microscopy (dSTORM)), the possibility to visualize the biological membranes and the preservation of the cellular ultrastructure by transmission electron microscopy (TEM) remains unsurpassed [1]. However, for an optimal imaging quality allowing the resolution of structures in the nanometer range, high requirements for tissue preservation have to be met. Aside from rare suitable brain biopsies or surgical specimens, histological studies of human central nervous system (CNS) diseases almost entirely rely on post mortem samples. Due to varying degrees of tissue degradation up until fixation, pathological changes can easily be obscured and achieving sufficient sample quality remains a challenging task [2]. On the other hand, animal models allow perfect control over the modalities of sacrifice, perfusion, and tissue sampling to obtain the best ultrastructural quality. However, genetic animal models can only be obtained for diseases with known mutations that exclude disorders with complex modes of inheritance (e.g., essential tremor [3]). Reprogramming of easily obtainable human cell types, such as fibroblasts, to either pluripotent stem cells [4], followed by differentiation into neurons [5] or to neural stem cells/neurons directly [6,7] allows the derivation of neurons from patients with a wide spectrum of diseases in vitro. Neurons in cell culture can readily be fixed and processed in ways suitable for TEM [8]. We previously published a rather uncomplicated, straight forward protocol based on plasmid transfection that provides directly reprogrammed human induced neural stem cells (iNSC) [9]. These cells can be cultured for many passages and differentiated into neurons or into astrocytes within one month. We, therefore, consider this protocol as a quite accessible way of obtaining neural cell types in vitro from healthy donors or patients. In this scientific study, we explored the ultrastructural preservation of neurons differentiated from iNSC under different culture conditions followed by fixation and TEM imaging of relevant neural structures. Furthermore, we compared different contrasting protocols in their ability to provide optimal visualization of the synaptic apparatus and other neuronal cell components.

## 2. Results

### 2.1. Differentiation of iNSC under Three Distinct Culture Conditions

Differentiation of neural stem cells into adult neurons can be achieved under various culture conditions. We tested three of them to find out which one offered the best ultrastructural preservation for TEM.

**Figure 1:**
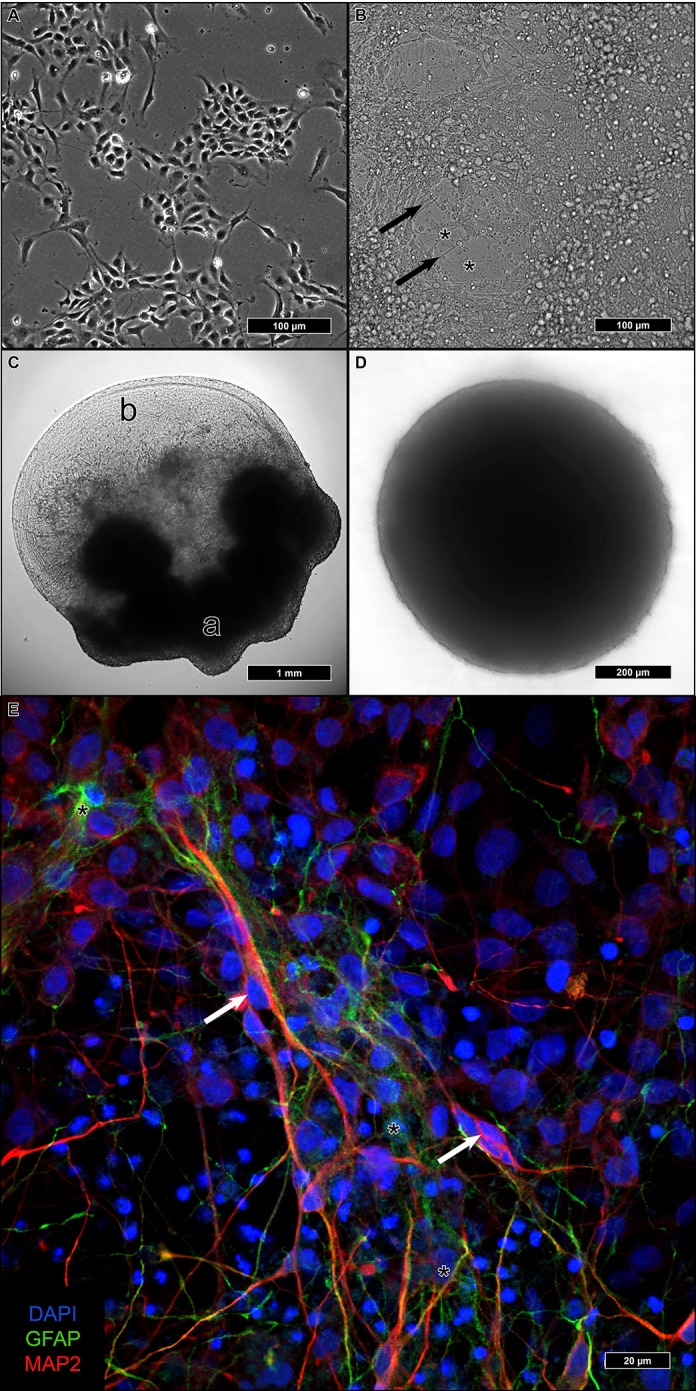
The three differentiation modalities in culture: induced neural stem cells (iNSC) growing adherently as a homogenous monolayer (**A**). After one month of adherent differentiation, neurite spreading cells (arrows) are placed on scattered layers of flat, presumably glial cells (asterisks) (**B**). Neural aggregates embedded in Matrigel, reaching several millimeters in size, kept their original polarity: spheres did not migrate away from their original site and form an apical part (a), while outgrowing neurites formed a dense network in the basal (b) parts of the aggregates (**C**). Neurospheres full of tightly packed cells appeared homogenously with a smooth surface, their size not exceeding 1 mm (**D**). Neurospheres replated on coverslips for outgrowth and incubated for immunofluorescence exhibited microtubule-associated protein 2 (MAP2)-positive neuronal (red, arrows) and glial fibrillary acidic protein (GFAP)-positive astrocytic cells (green, asterisks) in a proportion of roughly 5:1 (neurons: astrocytes) (**E**).

Adherent differentiation on coated coverslips started from 2D-cultured iNSC (Figure 1A). After reaching confluency, differentiation was initiated, and during the following four weeks, cells with a higher cytoplasma/nucleus ratio formed a basal layer with a dense network of neurite-sprouting cells on top (Figure 1B). In a previous study, we identified the first type as astrocytes and the second as neurons [9]. 

Aggregated iNSC in Matrigel remained at the embedding site and formed a dense fiber network spanning the entire droplet, which was several millimeters in size (Figure 1C). 

In u-shaped wells, iNSC aggregated within one to two weeks to a single compact sphere (Figure 1D). After the addition of a differentiation medium, the size of the spheres remained constant. In contrast to the considerable size of the Matrigel droplets, individual neurospheres remained below 1 mm in diameter. No discernible changes in the spheres were visible upon differentiation.

After re-plating neurospheres on coated glass coverslips, attachment of the spheres and outgrowth of cells could be observed (Figure 1E). Immunofluorescence could identify both microtubule-associated protein 2 (MAP2)-positive neurons as well as glial fibrillary acidic protein (GFAP)-positive astrocytes with a proportion of 5:1 (neurons:astrocytes).

### 2.2. Ultrastructural Preservation under Three Different Culture Conditions

INSC, differentiated under the described culture conditions, underwent fixation, contrastation, and embedding following standard protocols. 

**Figure 2:**
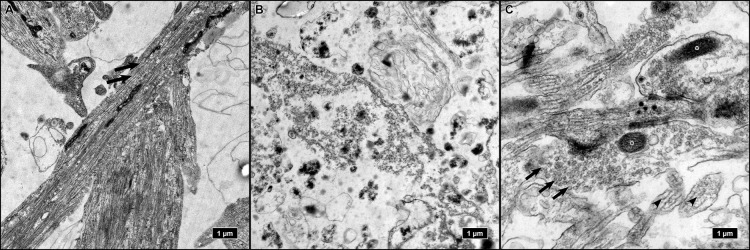
Ultrastructure of the three differentiation modalities: Adherent differentiation on coverslips resulted in the loss of all neuronal cells after sample preparation for transmission electron microscopy (TEM). Only astrocytic processes with intermediate filaments (arrows) were preserved (**A**). Spheres embedded in Matrigel exhibited a fragmented ultrastructure with low preservation of integrity (**B**). Only neurospheres provided preservation of neuronal elements, such as synapses, with vesicles (arrows), and synaptic mitochondria (asterisks), and neurites with neurofilaments (arrowheads) (**C**).

Cells on coverslips, despite remaining adherent during differentiation, tended to lift off during the preparation for TEM. When specimens were imaged by TEM, only processes containing intermediate filaments, and thus, most likely belonging to the astrocytic basal layer, remained (Figure 2A, Supplementary Figure S1). The ultrastructure was decently preserved, but no traces of neuronal cells (such as neurofilaments or synapses) could be found.

iNSC embedded and differentiated in Matrigel droplets exhibited inferior preservation of subcellular structures. Membranes and intracellular elements obviously had lost their integrity during sample processing for TEM and appeared fragmented (Figure 2B).

The only culture condition that provided sufficient ultrastructural preservation for TEM analysis was the neurosphere culture. Neurospheres remained tightly packed during fixation and embedding. The high cell density inside the spheres allowed screening and visualization of a high number of features in a small area (Figure 2C). 

Since only neurospheres provided a sufficient ultrustructural quality after fixation and embedding, we settled on this differentiation method for further analyses. 

### 2.3. Comparison of Three EM Preparation Protocols

Neuronal tissue staining for analysis of synaptic connections by electron microscopy requires optimal ultrastructural preservation in combination with strong deposition of heavy metal compounds into the biological membranes that outline neuronal processes, including axons and dendrites as well as synaptic vesicles. Besides combined primary fixation using a combination of glutaraldehyde and formaldehyde, several other parameters, including pH, osmolarity, and temperature of the washing buffer and primary fixative, are important for the success of ultrastructural preservation of neuronal tissues. In all three protocols, we used cacodylate buffer or phosphate buffer which have both been shown to be highly suitable for the preservation of neuronal tissue.

To enhance membrane contrast, standard staining protocols, including our standard-stain protocol, mostly used a combination of Osmium (Os) tetroxide (OsO_4_), and uranyl acetate (UA).

In the second protocol, we added the inorganic dye Ruthenium red (ammoniated ruthenium oxy-chloride) to OsO_4_ to enhance the staining, as it has been shown that when used in combination the two compounds react to form ruthenium tetroxide, which reacts with several cellular components resulting in enhanced contrast of diverse tissues [10,11]. 

Our third protocol was based on a study published by Deerinck and colleagues (Deerinck et al., 2010), which was designed primarily to emphasize the contrast of cellular membranes for serial block-face electron microscopy. Our high contrast en-bloc staining protocol combined subsequent steps after primary aldehyde fixation, including ferrocyanide-reduced osmium tetroxide postfixation, thiocarbohydrazide-osmium liganding (OTO), and subsequent uranyl acetate and en bloc lead aspartate staining. As Ca^2+^ ions are known to enhance membrane preservation and staining, CaCl_2_ was included in a number of steps.

These three EM preparation protocols (standard stain, Ruthenium red stain, and high contrast en-bloc stain) were compared with respect to ultrastructural preservation and optimal visualization of biological membranes of the synaptic apparatus and other neuronal cell components. 

**Figure 3:**
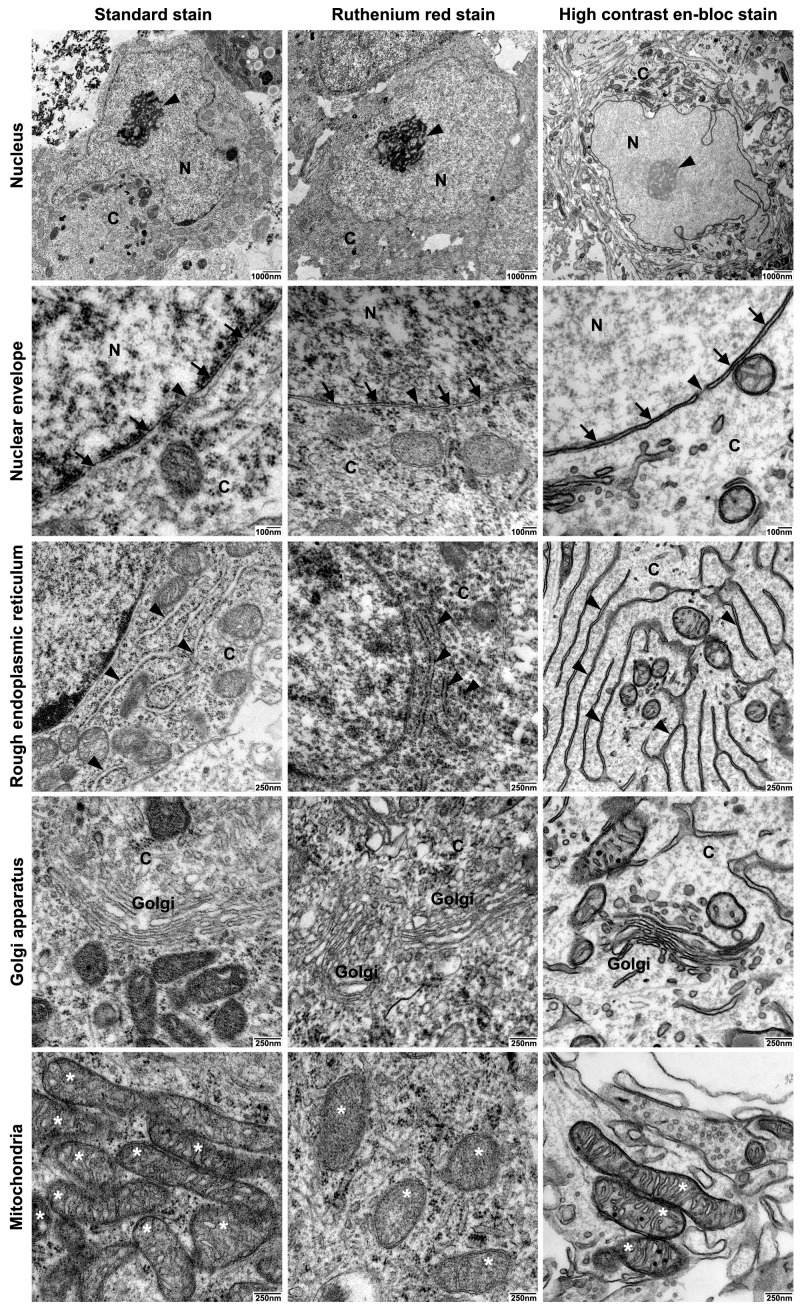
Comparison of the three fixation and staining protocols: Neuronal soma. Three TEM preparation protocols were compared with respect to ultrastructural preservation and optimal visualization of biological membranes of neuronal somata and cellular organelles: Transmission electron micrographs from cultured iNSC-derived neurospheres prepared using standard TEM staining protocol (standard stain, left column), Ruthenium red staining protocol (Ruthenium red stain, middle column), or high contrast electron microscopic staining protocol (high contrast en-bloc stain, right column). Panel 1: Neuronal soma. Soma showing cell nuclei (N) and nucleoli (arrowhead). Note the strong contrast of cellular membranes after sample processing with high contrast en-bloc staining protocol. C: Cytoplasm. Scale bars: 1000 nm. Panel 2: Nuclear envelope and nuclear pores. Preservation of the nuclear envelope (arrows) was improved by sample processing with high contrast en-bloc stain, which resulted in intact and essentially parallel double membranes with clearly visible nuclear pores (arrowhead). N: nucleus, C: Cytoplasm. Scale bars: 100 nm. Panel 3: Rough endoplasmic reticulum. Optimal preservation and visualization of membranes of rough endoplasmic reticulum with flattened cisternae uniformly arranged in long profiles (arrowheads) after sample processing with high contrast en-bloc stain. Note that ribosomes are less visible after sample processing with high contrast en-bloc stain when compared to samples processed with standard stain or Ruthenium red stain. C: Cytoplasm. Scale bars: 250 nm. Panel 4: Golgi apparatus. Optimal preservation and visualization of golgi membranes with flattened cisternae uniformly arranged in long profiles and golgi vesicles after sample processing with high contrast en-bloc stain. C: Cytoplasm. Scale bars: 250 nm. Panel 5: Mitochondria (marked by asterisks). Optimal preservation and visualization of mitochondria with clearly visible outer double membrane, intact cristae, dense matrix, and mitochondrial granules after sample processing with high contrast en-bloc stain. Scale bars: 250 nm.

All relevant subcellular structures were clearly discernible by all three protocols. However, unlike the neurospheres processed by the high contrast en-bloc stain, biological membranes were often not clearly visible in the neurospheres processed by the other two protocols (Figure 3–panel 1). We noticed that the chromatin was weakly stained in the high contrast en-bloc stain-processed specimens, likely due to the presence of membrane enhancing reagents during sample preparation (Figure 3–panel 1).

Detailed analysis of subcellular components in the differentially processed samples revealed that the continuity of nuclear envelope, and nuclear pores were preserved to a lesser extent in standard- or Ruthenium red stain-treated samples, whereas optimal preservation was achieved in the high contrast en-bloc stain-treated samples (Figure 3–panel 2). Membranes were essentially parallel to each other and showed no breaks and nuclear pores were clearly visible. No artificial dilation of the intermembraneous space of the nuclear envelope (Figure 3–panel 2), the rough endoplasmic reticulum (Figure 3–panel 3), or the golgi apparatus (Figure 3–panel 4) was seen in the high contrast en-bloc stain-treated samples compared to standard stain-treated samples. Dilation was seen to a lesser extent in Ruthenium red stain-treated samples (Figure 3–panel 2, panel 3, panel 4). However, ribosomes of the outer nuclear membrane, rough endoplasmic reticulum as well as free ribosomes could only be seen clearly in standard- or Ruthenium red stain-treated samples (Figure 3–panel 2, panel 3). Visualization of free ribosomes could be improved using post-staining of TEM sections with prolonged incubation with UAR (uranyl acetate replacement stain, Supplementary Figure S2). Analysis of all samples processed according to the three different protocols revealed sufficient preservation of mitochondria with only mild shrinkage or swelling observed in the neurospheres (Figure 3–panel 5). However, the double membranes and the cristae of mitochondria were only visible as continuous and undilated structures in the high contrast en-bloc stain-treated samples (Figure 3–panel 5).

**Figure 4:**
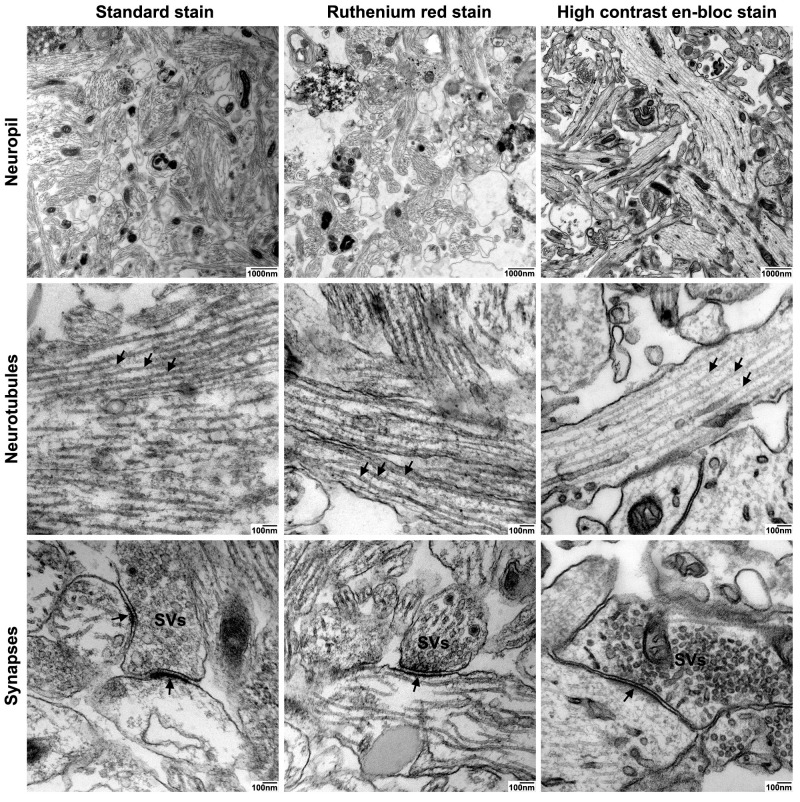
Comparison of the three fixation and staining protocols: Neuropil. Three TEM preparation protocols were compared with respect to ultrastructural preservation and optimal visualization of neuropil: Transmission electron micrographs from cultured iNSC-derived neurospheres prepared using standard TEM staining protocol (standard stain, left column), Ruthenium red staining protocol (Ruthenium red stain, middle column), or high contrast electron microscopic staining protocol (high contrast en-bloc stain, right column). Panel 1: Neuropil. Overview of neuronal processes. Note the strong contrast of cellular membranes after sample processing with high contrast en-bloc stain. Scale bars: 1000 nm. Panel 2: Neurotubules. Higher magnification of neuropil showing preservation of neurotubles (arrowheads) in neuronal processes after sample preparation using the different protocols. Scale bars: 100 nm. Panel 3: Synapses. Optimal preservation and visualization of membranes of the pre- and postsynapse as well as synaptic vesicles (SVs) after sample processing with high contrast en-bloc stain. Note the optimal preservation of the postsynaptic density (arrow) after sample processing with high contrast en-bloc stain. Scale bars: 100 nm.

**Figure 5:**
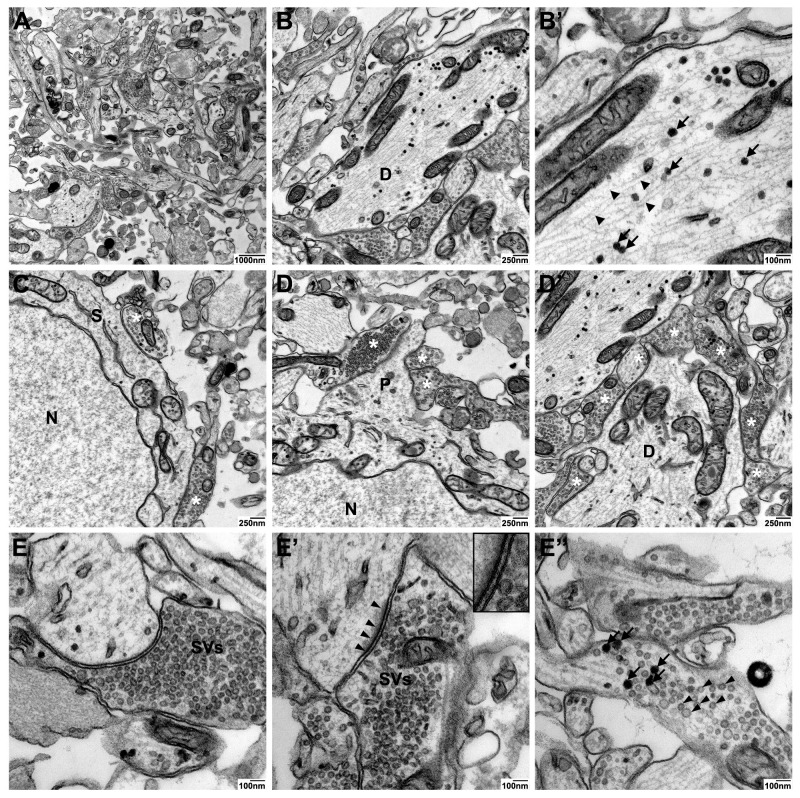
High contrast en-bloc staining protocol offers high-quality ultrastructural preservation and optimal membrane visualization in cultures neurospheres: Synapses. Transmission electron micrographs from cultured iNSC-derived neurospheres prepared using high contrast electron microscopic staining (high contrast en-bloc stain). Overview of neuronal processes (**A**). Single neuronal process (dendrite, **D**) at lower (**B**) and higher magnification (**B’**) showing neurofilaments (arrowheads) and dense core vesicles (arrow) with optimal preservation of cellular membranes. Axosomatic synapses (**C**) and axodendritic synapses (**D**, **D**’); presynaptic compartments. Examples of individual synapses (**E**–**E**’) showing optimal preservation of pre- and postsynaptic membranes, small synaptic cleft, postsynaptic density (**E**’, arrowheads), and synaptic vesicles (SVs). Enlarged view of the boxed region in E’ showing optimal preservation and staining of the lipid bilayers of pre- and postsynaptic membranes. Different types of synaptic vesicles could clearly be distinguished in neuronal processes showing clear (arrowheads) and dense core (arrows) vesicles (**E**’’). N: nucleus, S: soma, P: process. Scale bars as indicated.

The particularly enhanced membrane contrast in high contrast en-bloc stain-treated neurospheres led to optimal preservation and discernability of axonal and dendritic processes in the neuropil (Figure 4–panel 1, panel 2). In all three protocols, neurotubules were preserved, but neuronal membrane visualization was highly improved by high contrast en-bloc staining (Figure 4–panel 2). In addition, this method offers high-quality ultrastructural preservation and excellent membrane staining of synaptic connections, although the postsynaptic density was stained less intensely (Figure 5E’) compared to those processed with standard- or Ruthenium red stain (Figure 4–panel 3). Since UA has been implied in labeling proteins of the postsynaptic density similar to heterochromatin, we speculate that the weaker staining of these structures in our high contrast en-bloc stain-processed neurospheres is a result of interference with other membrane enhancing reagents present during sample preparation.

Detailed analyses of neuronal and synaptic structures in high contrast en-bloc stain-processed neurospheres revealed high-quality ultrastructural preservation and excellent membrane staining of dendritic processes with neurofilaments, dense core vesicles as well as axosomatic and –dendritic synapses (Figure 5A–D). A particular strength of the high contrast en-bloc stain was observed for visualizing subcomponents of the synaptic apparatus: The very clear membrane contrast made the pre- and postsynaptic membrane with the interjacent synaptic cleft easily discernible (Figure 5E and E’). Different types of synaptic vesicles (clear vs. dense core), docked vesicles at the presynaptic membrance, or free vesicles of the resting pool (Figure 5E–E’’) could be clearly visualized. 

**Figure 6:**
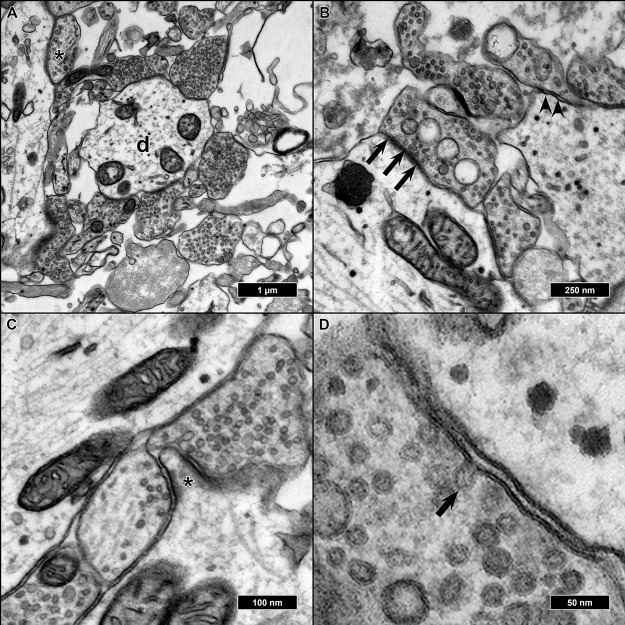
EM micrographs of features associated with synaptic maturity: (**A**) Synaptic boutons terminating at a dendrite (d). All entirely filled with synaptic vesicles but one (asterisk) which showed a sparser filling and thus exhibited a lesser state of maturity. (**B**) The majority of synapses were asymmetric (arrows), and some of them symmetric (arrowheads). (**C**) Protrusions resembling dendritic spines (asterisk) with surrounding synaptic boutons, but absent spine apparatus. (**D**) Synaptic bouton with a vesicle in the moment of fusion with the presynaptic membrane (arrow) as a sign of synaptic functionality.

Features of advanced synaptic maturity could be observed in the analyzed samples: All synapses appeared as entirely or nearly filled with synaptic vesicles. We could never observe synapses with single or no vesicles inside (Figure 6A). Of all synaptic contacts, 75% appeared as asymmetric synapses (postsynaptic membrane appearing thicker and more contrasted than the presynatic) (Figure 6B). As already described, most synapses were either axo-dendritic or axo-somatic, sporadically synaptic contacts could also be found on protrusions from the main dendrite, presumably representing spines, but without smooth endoplasmatic reticulum present inside the protrusions (Figure 6C). Occasionally, cells were fixed in the very moment of fusion between a synaptic vesicle and presynaptic membrane as a morphological correlate of synaptic transmission (Figure 6D).

## 3. Discussion

For decades visualizing synaptic structures and studying their morphology has been a particular strength of the TEM. In the past, the general notion has been that synaptic transmission is highly conserved [12] and thus was studied in a wider range of model organisms both invertebrate and vertebrate. However, the presence of distinctive features in the human neuromuscular synapse [13] and striking differences in the postsynaptic human proteome in comparison to mouse [14] challenge this notion. Therefore, there are obvious reasons to study synaptic neurotransmission in human neurons, but the aforementioned limitations of sufficiently preserved human CNS tissue challenge these endeavors. Reprogramming techniques can provide human neurons of healthy controls and patients with different kinds of diseases in vitro [15]. After a sufficient time span of differentiation, a functional synaptic network is established [9]. There have been studies in the past, which demonstrated the presence of synaptic connections between human neurons derived from reprogrammed stem cells by electron microscopy on a proof-of-principle basis [8]. However, a systematic comparison of differentiation and TEM preparation protocols concerning optimal visualization of neuronal elements of these cells has not been performed. 

The first (and until today, most employed) reprogramming paradigm is the reprogramming towards pluripotent stem cells [4]. Most studies that had a more detailed look into the synapse and neuronal network formation of human neurons in vitro used this cell type [16]. These cells and their pluripotency are maintained by complex and work-intensive protocols requiring almost daily media change and manual removal of spontaneously differentiated cells. For deriving mature neurons, multi-step protocols, combining neural induction, regional patterning, and terminal differentiation, have to be followed that can easily take several months [15]. While this allows a certain enrichment of desired cell types, a 100% pure cell type is never achieved. Adherent differentiation on coated coverslips has been the standard approach leading to immature synapse formation after less than one week and spontaneous synaptic activity after roughly one month [17]. However, a full maturity (e.g., formation of synapses on spines) is possibly not achieved. Neuralizing and differentiating pluripotent stem cells as 3D aggregates (organoids) results in a maturation over months and is more likely to result in a mature synaptic network [18]. In our personal opinion, a culture technique requiring many months until specimens can be studied poses severe challenges to planning and performing experiments (especially if replicates are required).

The alternative is the generation of induced neurons (iN) from somatic cells: Overexpressing transcription factors associated with neuronal identity can reprogram non-neural cells to neurons [19]. The time needed for the reprogramming process to be completed is of similar length as the time needed for neural differentiation from induced pluripotent stem cells (iPSC) [20]. However, since iN are post-mitotic, the number derived is rather small and cannot be increased by proliferation. Thus, the reprogramming process has to be repeated for every set of new experiments.

A good compromise, in our opinion, is induced neural stem cells (iNSC) employed in this study: Derived from somatic cells by plasmid-based transfection, they are proliferative for at least 25 passages but cultivating them requires much less intervention and they are not prone to spontaneous differentiation [9,21]. Differentiation is simply initiated by a change in cell culture media and the addition of three recombinant growth factors and one small molecule. The timing until acquiring a certain level of maturity is not different from the other two methods described. The biggest disadvantage is that these cells are not responsive to patterning cues, meaning their regional identity cannot be altered. They exhibit a quite stable mix of neuronal subtypes (60% upper layer cortical layer, 20% GABAergic, 20% dopaminergic). Therefore, they might not be the first choice when it comes to obtaining a specific cell type, but their strength lies more in providing a good mixture of different neuronal subtypes in one dish.

Another important question in this context is the maturity and functionality of the synaptic network derived from reprogrammed stem cells. Neurons derived from iPSC might appear mature by morphology or specific protein expression after a couple of weeks in culture, yet the formation of functional networks usually takes more than a month, and still not all electrophysiological features associated with them might be present [16]. The same seems to apply to synaptic contacts. Morphologically, synapses begin their existence as mere contacts between two neuronal membranes. Later synaptic vesicles fill the presynaptic bouton, the pre- and postsynaptic membrane becomes more and more defined (resulting in an increasing number of asymmetric synapses with a thicker postsynaptic membrane) and an active synapse capable of neurotransmitter release by fusion of the synaptic vesicles with the presynaptic membrane has come into existence [22,23]. Certain neurons form dendritic spines, highly dynamic structures for multiple synaptic contacts [24]. The timing and sequence of synaptogenesis have already been studied in the human fetus decades ago, but only recently it has been acknowledged that neurons derived from human stem cells in vitro exhibit a different timing in synaptogenesis and certain features associated with synaptic maturity (e.g., spine formation) might be absent altogether [17]. As usual, when dealing with stem cell-derived neurons, much is dependent on the individual protocol employed. The neurospheres from iNSC we employed in our study exhibited a particularly mature phenotype: The majority of synaptic boutons were densely filled with synaptic vesicles, and the majority exhibited an asymmetric morphology and due to the superior membrane contrast of the high contrast en-bloc stain, individual fusions between vesicles and the presynaptic membrane could be observed. We even observed membrane protrusions that could be dendritic spines, a feature that has only be observed in iPSC derived cerebral organoids after many months in culture [25]. However, concerning this finding, uncertainty remained. We did not perform serial block-face scanning electron microscopy for three-dimensional reconstruction of the protrusions, which is the method of choice for unequivocally visualizing dendritic spines. Furthermore, we did not find a spine apparatus (smooth endoplasmatic reticulum inside the spine) in any protrusion. Not all spines contain a synaptic apparatus, but the presence of it is generally considered a sign of maturity [26]. We would, therefore, consider the presence of possibly still immature spines with no signs of full maturity. 

The iNSC employed in this study were cultured adherently on Matrigel. In a previous publication, we induced differentiation of iNSC into mature neurons on glass coverslips as it simplified staining and fluorescence microscopy, transfer to recording chambers, etc. [9]. As we observed in the past, iNSC generated using our protocol differentiate into both neurons and astrocytes with the neurons exhibiting the tendency to aggregate on top of the astrocytes (Figure 1B). Presumably, the numerous pipetting steps leading to repeated shear stress resulted in a detachment of the neuronal layer leaving only the astrocytic basal layer. 

Aggregating iNSC into neurospheres and embedding them into Matrigel droplets followed a protocol similar to the formation of cerebral organoids [18]. However, instead of forming a compact neuroepithelial layer with ventricle-like cavities, differentiating iNSC remained stationary and extended neurites throughout the aggregates. In contrast to iPSC-derived embryoid bodies that served as seeds for the cerebral organoids, our iNSC were different from early neuroepithelial precursors. We can only speculate that the comparatively large size of the aggregates of a few millimeters in conjunction with being mainly composed of a neurite fiber network, led to insufficient preservation for TEM.

Only the densely packed and rather small neurospheres reliably provided a sufficient ultrastructure. Neurospheres were generated by culturing iNSC under growth conditions until single spheres formed [27,28]. The growth of the spheres ceased after the induction of differentiation. Thus, the size of the spheres was mainly determined by the cell number seeded per well. This provided a good compromise between a small size allowing sufficient penetration of the fixatives and solutions during the embedding process and safe handling under the naked eye. Furthermore, the small size, yet a high density of cell bodies and processes, simplified screening during TEM analysis for relevant structures. 

Besides the ultrastructural preservation, reliably identifying structures of interest by TEM requires high contrast with a sharp delineation. Neuronal structures that usually receive the most attention in TEM studies are either membrane-rich as synapses and mitochondria or filamentous, such as neurofilaments or neurotubuli. The standard- and Ruthenium red-stainings appeared “grainy”, and the contours of membranes were sometimes hard to discriminate from surrounding structures. A better contrast was achieved for structures rich in DNA or RNA (nucleus, ribosomes) or proteins (filaments and tubuli). The high contrast en-block stain was primarily designed for enhancing membrane contrast in mammalian tissue in serial block-face scanning electron microscopy (SBF-SEM) [29]. Although developed for a different EM method, preserving and contrasting membranes proved useful for TEM as well. En-bloc contrasting protocols were optimized for better penetration of larger tissue samples and superseded the contrasting of ultra-thin cut sections on grids which is prone to non-specific deposition of the contrasting heavy metals [30]. In contrast to the other two staining protocols tested here, CaCl_2_ (which is known to improve the stability of lipid bilayers [31]) was added to the fixative as well as to some of the contrasting steps. The increased contrast of membranes with this protocol was the result of a combination of different staining principles established in numerous individual studies: Ferrocyanide reduced osmium post-fixation in combination with cacodylate buffer, partially extracted the cytoplasmatic ground substance and mitochondrial matrix while strongly binding to membranes [32]. Insufficient preservation of the lipid bilayer structure by the aforementioned method was compensated by a downstream osmium–thiocarbohydrazide–osmium (OTO) step [33]. Using the classical contrasting agent uranyl–acetate (highly toxic and nowadays replaced by samarium and gadolinium [34]) and lead aspartate in pre-embedding en-bloc staining of wet tissue overcame their tendency to form contaminating precipitates when applied post-embedding [35,36].

To our knowledge, this is the first systematic comparison of different protocols for the visualization of neurons derived from human reprogrammed cells by TEM and the first example of en-bloc staining techniques employed on three-dimensional cellular aggregates. Consequently, the paramount properties of en-bloc staining in the preservation and contrasting of neuronal membranes work for in vitro specimens as well.

## 4. Materials and Methods

### 4.1. Derivation and Proliferation of iNSC

Detailed protocols describing the reprogramming and culture of iNSC from human fibroblast cultures have been published before [9,21]. In short, fibroblast cultures from 2 healthy donors who gave informed consent following the requirements and positive votum (AZ12-219) of the ethics committee of the University of Lübeck, Germany, were transfected with three polycistronic plasmids overexpressing transcription factors associated with pluripotency (Oct3/4, Sox2, Klf4, L-myc, Lin28) as well as a small hairpin RNA directed against p53 [37]. The presence of Epstein–Barr virus (EBV)-derived origin of viral replication/Epstein–Barr virus nuclear antigen 1 (oriP/EBNA-1) ensured the DNA amplification during cell cycles and extended the presence of plasmids in the cells. After switching the standard fibroblast medium (DMEM, 10% fetal calf serum, 1% Glutamax, 1% Antibiotic/Antimycotic, all from Thermo Fisher Scientific, Waltham, USA) to a commercially available neural induction medium (STEMCELL Technologies, Vancouver, Canada). Neural colonies began to emerge after about two to three weeks, were mechanically picked, and replated on Matrigel (Thermo Fisher Scientific, Waltham, USA) coated culture dishes. The growth medium was changed to neural progenitor medium (STEMCELL Technologies, Vancouver, Canada), confluent cultures split by accutase (Thermo Fisher Scientific, Waltham, USA) digestion and replated in a ratio of 1:10 for continuous proliferation. 

### 4.2. Differentiation of iNSC

For differentiation, iNSC were split and viable cells determined by staining with 1:10 Trypan Blue 0.4% (Thermo Fisher Scientific, Waltham, MA, USA) and counting with a Neubauer improved counting chamber (Glaswarenfabrik Karl Hecht GmbH & Co KG, Sondheim vor der Rhön, Germany). INSC were plated and differentiated following three different protocols. All experiments were performed with the two iNSC lines in duplicates.

#### 4.2.1. Adherent Differentiation on Glass Coverslips

Twelve-millimeter glass coverslips (Glaswarenfabrik Karl Hecht GmbH & Co KG, Sondheim vor der Rhön, Germany) were surface treated by 65% sulphuric acid (Carl Roth, Karlsruhe, Germany) overnight and heated to 180° for 8 h. Once put inside 24-well culture plates (Sigma–Aldrich, St. Louis, MI, USA), we performed coating overnight with a poly-D-lysine (Sigma–Aldrich, St. Louis, USA) solution (0.075 mg/mL in 0.1 M borate buffer) at room temperature and overnight by a laminin (Sigma–Aldrich, St. Louis, USA) solution (5 µg/mL in PBS) at 37 °C. One hundred and fifty thousand cells per well were plated in a neural progenitor medium with the addition of 1 µM of the rho kinase inhibitor Y-27632 (STEMCELL Technologies, Vancouver, Canada). After iNSC reached confluence, differentiation started by switching to a neural differentiation medium, composed as follows: DMEM/F12:Neurobasal 1:1, 1% N2, 2% B27 (Gibco by Life Technologies, Vancouver, BC, Canada), 20 ng/mL brain-derived neurotrophic factor (BDNF), 10 ng/mL glial cell line-derived neurotrophic factor (GDNF), 10 ng/mL insulin-like growth factor 1 (IGF-1) (all from PeproTech, Rocky Hill, NJ, USA), 0,5mM dibutyril cyclic adenosine–monophosphate (dbcAMP, EnzoLife Sciences, Farmingdale, NY, USA) and 10 μM of the Notch-pathway inhibitor DAPT (Tocris, Ellisville, MO, USA). Differentiation continued for four weeks.

#### 4.2.2. Free Floating Differentiation of Aggregates Embedded in Matrigel

The second differentiation protocol was adapted from a protocol of cerebral organoid formation [18]: Fifty thousand cells per well were plated inside a u-shaped ultra-low attachment 96-well-plated (Thermo Fisher Scientific, Waltham, MA, USA) in neural progenitor medium. Cells aggregated into spheres for 7 days, were removed from the wells and embedded in groups of 5 inside 50 µL droplets of ice-cold Matrigel (Thermo Fisher Scientific, Waltham, MA, USA) on a sheet of sterilized parafilm (Bemis, Neenah, WI, USA). After polymerization for 30′ at 37 °C, the droplets were removed from the film, transferred into ultra-low attachment 6-well plates (Thermo Fisher Scientific, Waltham, MA, USA) and differentiation induced by differentiation medium for four weeks.

#### 4.2.3. Free Floating Differentiation of Neurospheres

This method corresponded to the method described under 4.2.1 except that cells were kept and differentiated inside the ultra-low attachment 96-well plates for four weeks.

#### 4.2.4. Replating and Immunofluorescence of Neurospheres

After four weeks of differentiation, neurospheres were put onto coverslips coated as described under 4.2.1 in the same medium as described above. Neurospheres attached to the coated surface and cells started migrating out. After one week, spheres and cells were fixed with 4% Paraformaldehyde (Carl Roth, Karlsruhe, Germany) and washed with phosphate-buffered saline (Thermo Fisher Scientific, Waltham, MA, USA). After blocking of unspecific binding sites with 5% donkey serum in PBS with 0.1% Triton X-100 (Thermo Fisher Scientific, Waltham, MA, USA), primary antibodies (MAP2, Millipore, Cat. No. MAB378, 1:500 and GFAP Zytomed Systems Cat. No. RBK037 1:500) were incubated overnight at 4 °C in 1% donkey serum in PBS with 0.1% Triton X-100. The next day specimens were washed for 3 × 15 min in PBS + 0.1% Triton X-100, followed by an incubation of the secondary antibodies (donkey anti-mouse CF568 and donkey anti-rabbit CF488, both Sigma–Aldrich, St. Louis, USA) for 2 h at room temperature. After another washing step for 3 × 15 min, nuclear staining wash performed by adding 1 µg/mL DAPI (Thermo Fisher Scientific, Waltham, USA) in PBS to the cells for 10 min and washing for 3 × 5 min with PBS. Coverslips were embedded on microscope slides (Glaswarenfabrik Karl Hecht GmbH & Co KG, Sondheim vor der Rhön, Germany) with ProLong Glass (Thermo Fisher Scientific, Waltham, MA, USA), and stored at 4 °C in the dark. Imaging was performed with an Olympus FV1000 confocal laser scanning microscope (Olympus, Tokyo, Japan). Six high-power fields (60× magnification) were taken and analyzed. 

### 4.3. Specimen Preparation for Transmission Electron Microscopy

#### 4.3.1. Standard Electron Microscopic Preparation (Standard Stain)

Coverslips, aggregates in Matrigel, or neurospheres were washed to remove cell culture medium and fixed in 0.1 M cacodylate buffer pH 7.4 containing 2.5% glutaraldehyde, 4% formaldehyde (fresh from paraformaldehyde) for 3 h at room temperature (RT) and left overnight in a fixative at 4 °C. After washing for 3 × 10 min in cold PBS (phosphate-buffered saline, pH 7.2), samples were subsequently fixed for 1 hr with 1% osmium tetroxide (buffered with PBS, pH 7.2). They were washed 3 x 10 min in PBS and 1 x 10 min in ddH_2_O at RT. Afterward, specimens were incubated in aqueous UAR-EMS (4%, Uranyl acetate replacement stain, Electron Microscopy Sciences, Hatfield, USA). Washed 3 × 10 min in ddH_2_O at RT and dehydrated in an ascending ethanol series using solutions of 30%, 50%, 70%, 90%, 96%, 100%, 100% ethanol 10 min each. They were incubated two times in propylene oxide (PO) for 30 min each before incubation in a mixture of PO and Epon812 (1:1) overnight. The following day, the Epon-PO mixture was substituted with pure Epon812 and samples were incubated for 2 h in Epon812. Specimens were embedded in Epon812 and kept at 60 °C for 48hrs. For ultrathin sections, 70 nm thick ultrathin sections were cut with an ultramicrotome (Ultracut E, Reichert Jung, Germany) and collected on copper or nickel grids. Sections were post-stained with aqueous UAR-EMS (2,5%, Uranyl acetate replacement stain, Electron Microscopy Sciences, Hatfield, USA) and 0.2% lead citrate and finally analyzed with an LEO AB 912 transmission electron microscope (Carl Zeiss Microscopy GmbH, Germany). 

#### 4.3.2. Electron Microscopic Preparation Using Ruthenium Red (Ruthenium Red Stain)

To provide enhanced contrast staining of neurospheres compared to conventional electron microscopic protocols, the polycationic stain Ruthenium red was used in combination with OsO4 staining as follows: Neurospheres were washed to remove cell culture medium and fixed in 0.1 M cacodylate buffer pH 7.4 containing 2.5% glutaraldehyde, 0.01% Ruthenium red for 3 h at RT and left overnight in fixative at 4 °C. After washing for 3 × 5 min in 0.1 M cacodylate buffer, neurospheres were subsequently fixed for 1 hr with 2% osmium tetroxide (buffered with 0.1 M cacodylate buffer, pH 7.2). Neurospheres were washed 3 × 5 min 0.1 M cacodylate buffer and 1 × 5 min in ddH_2_O at RT. Neurospheres were incubated in aqueous UAR-EMS (4%, Uranyl acetate replacement stain, Electron Microscopy Sciences, Hatfield, PA, USA), washed 3 × 5 min in ddH_2_O at RT and dehydrated in an ascending methanol series using solutions of 25%, 50%, 70%, 80%, 95%, 95%, 100%, 100% methanol 10 min each. Neurospheres were incubated two times in Ethoxy propanol for 10 min each and incubated for 2 h in Epon812. Specimens were embedded in Epon812 and kept at 60 °C for 48hrs. For ultrathin sections, 70 nm thick ultrathin sections were cut with an ultramicrotome (Ultracut E, Reichert Jung, Germany) and collected on copper or nickel grids. Sections were post-stained with aqueous UAR-EMS (2,5%, Uranyl acetate replacement stain, Electron Microscopy Sciences, Hatfield, USA) and 0.2% lead citrate and finally analyzed with an LEO AB 912 transmission electron microscope (Carl Zeiss Microscopy GmbH, Germany).

#### 4.3.3. High Contrast Electron Microscopic Preparation (High Contrast En-Bloc Stain)

To specifically enhance the staining of neuronal membranes, neurospheres were prepared using a modification of the National Center for Microscopy and Imaging Research (NCMIR) protocol [29], which was initially developed for the preparation of biological specimens for serial block-face scanning electron microscopy. Briefly, we rinsed neurospheres to remove cell culture medium and fixed in 0.12 M phosphate buffer (PB buffer) pH 7.5 containing 1.0% glutaraldehyde, 1.0% formaldehyde (fresh from paraformaldehyde), 0.002% CaCl_2_ and 2% sacharose for 3 h at RT and left overnight in a fixative at 4 °C. Subsequently, neurospheres were washed in 0.15 mM sodium cacodylate buffer (pH 7.4) containing 2 mM CaCl_2_ and incubated for 60 min on ice in a reduced osmium solution containing 2% osmium tetroxide, 1.5% potassium ferrocyanide, 2 mM CaCl_2_ in 0.15 mM sodium cacodylate buffer (pH 7.4). Neurospheres were washed in ddH_2_O at room temperature (RT) 5 × 5 min, followed by incubation in 1% thiocarbohydrazide (TCH) solution for 25 min at RT. Neurospheres were washed in ddH2O at RT, 5 × 5 min each and incubated in 2% osmium tetroxide in ddH_2_0 for 30 min at RT. Subsequently, neurospheres were washed 5 × 5 min at RT in ddH_2_O then incubated in aqueous 4% UAR-EMS. Neurospheres were washed 3 × 3 min in ddH_2_O at RT. Prior incubation with lead aspartate solution, neurospheres were washed 2 × 3 min in ddH_2_O at 60 °C, and then subjected to en bloc Walton’s lead aspartate staining [36] and placed in a 60 °C oven for 30 min. Neurospheres were washed 5 × 5 min in ddH_2_O at RT and dehydrated using ice-cold solutions of freshly prepared 30%, 50%, 70%, 90%, 100%, 100% ethanol (anhydrous), 100% acetone (anhydrous) for 10 min each, then placed in anhydrous ice-cold acetone and left at RT for 10 min. Neurospheres were placed in 100% acetone at RT for 10 min. During this time, Epon812 was prepared. The resin was mixed thoroughly and samples were placed into 25% Epon:75% acetone for 2 h, then into 50% Epon:50% acetone for 2 h and 75% Epon:25% acetone for 2 h. Neurospheres were placed in 100% Epon overnight. The next day, Epon was replaced with fresh Epon for 2 h and neurospheres were placed in beem capsules and incubated in a 60 °C oven for 48 h for resin polymerization. For ultrathin sections, 70 nm thick ultrathin sections were cut with an ultramicrotome (Ultracut E, Reichert Jung, Germany) and collected on copper or nickel gridsm, and finally analyzed with a LEO AB 912 transmission electron microscope (Carl Zeiss Microscopy GmbH, Germany).

## Supplementary Materials

Supplementary Materials can be found at https://www.mdpi.com/1422-0067/21/5/1708/s1.

## Abbreviations

TEM	Transmission electron microscopy
GA	Glutaraldehyde
dSTORM	Direct stochastic optical reconstruction microscopy
EBNA-1	Epstein–Barr virus nuclear antigen 1
EBV	Epstein–Barr virus
GFAP	Glial fibrillary acidic protein
iNSC	Induced neural stem cells
MAP2	Microtubule associated protein 2
OriP	Origin of viral replication
PFA	Paraformaldehyde
PO	Propylene oxide
RT	Room temperature
UAR	Uranyl acetate replacement
