# Supplementary material for: Visualizing the Synaptic and Cellular Ultrastructure in Neurons Differentiated from Human Induced Neural Stem Cells—An Optimized Protocol
Source: Int J Mol Sci. 2020 Mar 2;21(5):1708. doi: 10.3390/ijms21051708 (PMC7084184; doi:10.3390/ijms21051708)
Supplement: Supplementary file 1 [file ijms-21-01708-s001.pdf]

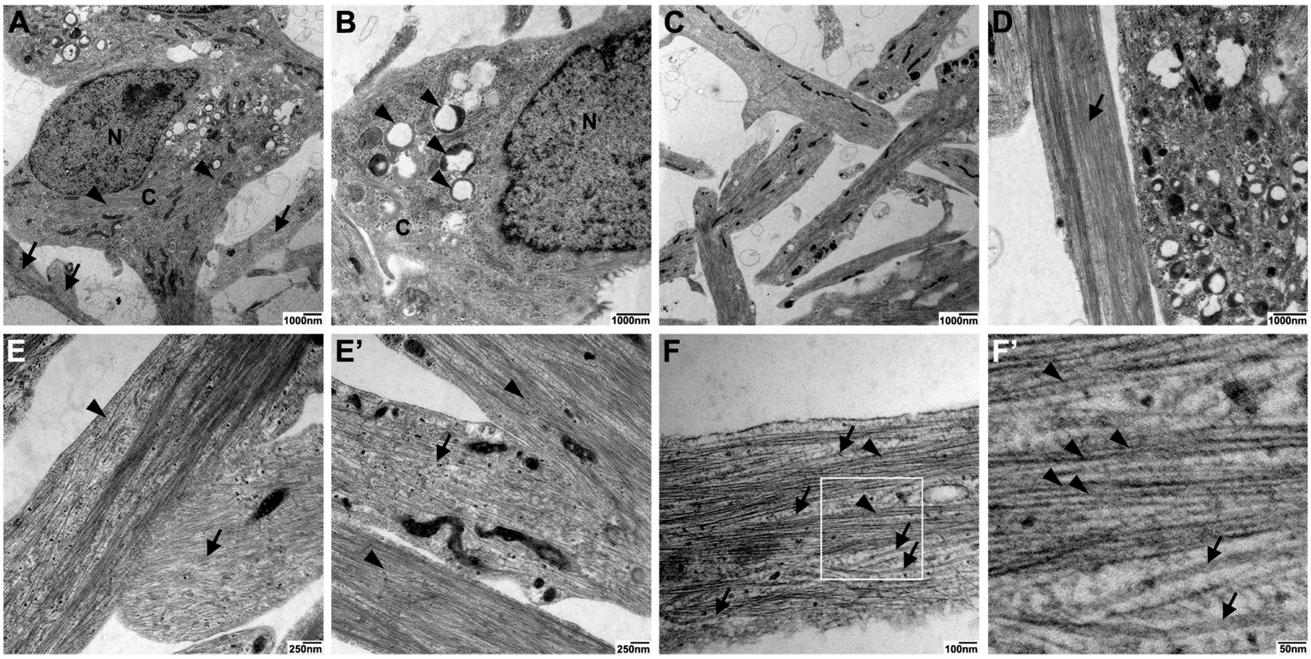

Supplementary Figure 1: Preservation of astrocytes and astrocytic processes

Transmission electron micrographs from adherent astrocytes on coverslips. Overview of astrocyte soma with bundles of intermediate filaments (arrowheads) and astrocytic processes (A, arrows), N: nucleus, C: cytoplasm. Scale bar: 1000nm. Higher magnification of astrocyte soma with hollow vesicular structures (B, arrowheads), note the high amount of heterochromatin, N: nucleus, C: cytoplasm. Scale bar: 1000nm. Overwiew of astrocytic processes (C), Scale bar: 1000nm. Astrocytic process (arrow) with densly packed and parallel oriented intermediate filaments (D), Scale bar: 1000nm. Two types of astrocytic processes can be distinguished (E, E’), processes with densly packed and parallel oriented intermediate filaments and sporadic microtubles (arrowheads) and processes with less densly packed and intermediate filaments, microtubles and more mitochondria (arrows). Scale bars: 250nm. Higher magnification of astrocytic processes (F, F’) with densly packed and parallel oriented intermediate filaments (arrowheads) and microtubles (arrows), Scale bars: 100nm, 50nm.

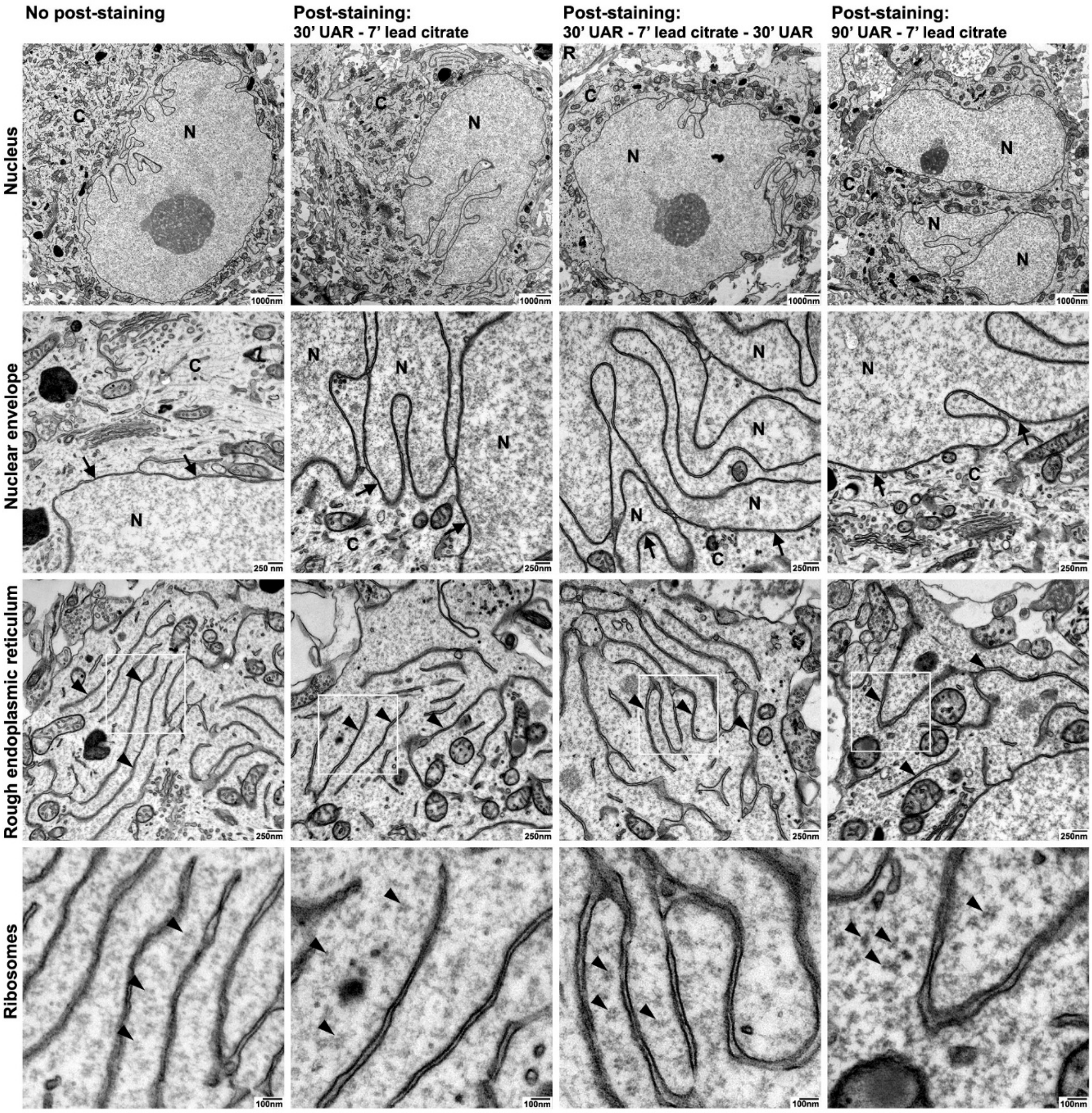

Supplementary Figure 2: Comparison of different post-staining protocols after sample processing by high contrast en-bloc stain to enhance visualization of ribosomes.

Transmission electron micrographs from cultured iNSC-derived neurospheres prepared with high contrast en-bloc stain only (left column); post-staining of sections with UAR, 30 min and lead citrate, 7min (second column); UAR, 30 min and lead citrate, 7min and UAR, 30 min (third column); UAR, 90 min and lead citrate, 7min (right column). All post-staining were performed using solutions of aqueous 2.5% uranyl acetate replacement stain and 0.2% lead citrate. Note that ribosomes are less visible after sample processing with high contrast en-bloc stain when compared to samples post-stained with UAR and lead citrate. Prolonged incubation with UAR (third column and right column) resulted in stronger staining of free ribosomes, while ER-attached ribosomes are still less visible when compared to conventionally-processed samples.

Panel 1: Neuronal soma. N: nucleus, C: cytoplasm. Scale bars: 1000nm.

Panel 2: Nuclear envelope (arrows). N: nucleus, C: cytoplasm. Scale bars: 250nm.

Panel 3: Rough endoplasmic reticulum (arrowheads). Scale bars: 250nm.

Panel 4: Higher magnifications (rough endoplasmic reticulum and free ribosomes) of boxed areas in panel 3. Scale bars: 100nm.
